# The DNA repair component Metnase regulates Chk1 stability

**DOI:** 10.1186/1747-1028-9-1

**Published:** 2014-07-09

**Authors:** Elizabeth A Williamson, Yuehan Wu, Sudha Singh, Michael Byrne, Justin Wray, Suk-Hee Lee, Jac A Nickoloff, Robert Hromas

**Affiliations:** 1 Department of Medicine, University of Florida College of Medicine, 1600 Archer Rd SW, Gainesville, FL 32610, USA; 2 Department of Biochemistry and Molecular Biology, Indiana University School of Medicine, Indianapolis, IN 46202, USA; 3 Department of Environmental and Radiological Health Sciences, Colorado State University, Fort Collins, CO 80523, USA

**Keywords:** DNA repair, Chk1, Ubiquitination, Cell cycle

## Abstract

Chk1 both arrests replication forks and enhances repair of DNA damage by phosphorylation of downstream effectors. Metnase (also termed SETMAR) is a SET histone methylase and transposase nuclease protein that promotes both DNA double strand break (DSB) repair and re-start of stalled replication forks. We previously found that Chk1 phosphorylation of Metnase on S495 enhanced its DNA DSB repair activity but decreased its ability to re-start stalled replication forks. Here we show that phosphorylated Metnase feeds back to increase the half-life of Chk1. Chk1 half-life is regulated by DDB1 targeting it to Cul4A for ubiquitination and destruction. Metnase decreases Chk1 interaction with DDB1, and decreases Chk1 ubiquitination. These data define a novel pathway for Chk1 regulation, whereby a target of Chk1, Metnase, feeds back to amplify Chk1 stability, and therefore enhance replication fork arrest.

## Findings

Metnase (also SETMAR) is a fusion of a SET [*Su(var)3-9, Enhancer-of-zeste, Trithorax*] domain and a transposase domain present only in anthropoid primates, and not in other mammals [1]. The Metnase SET domain di-methylates histone 3 lysine 36 (H3K36) [1], while the transposase domain has most but not all of the classic transposase nuclease activities, including 5’-terminal inverted repeats (TIR)-specific DNA binding, DNA looping, assembly of paired end complex (PEC), and DNA cleavage [1,2].

We found that Metnase enhances non-homologous end joining of DNA double strand breaks (DSB), perhaps via its association with DNA Ligase IV [1,3]. Both the histone methylase and nuclease domains are essential for Metnase-mediated DNA DSB repair [1]. The SET domain is responsible for di-methylating H3K36 adjacent to DNA DSBs, and this di-methylation stabilized the Ku and MRN complexes at the DSB [4]. The transposase nuclease domain can trim non-compatible free DNA ends at a DSB, and thereby improve their end-joining [5]. Metnase is also essential for proper re-starting of stalled replication forks, perhaps by nicking and decatenating positively supercoiled DNA in front of the fork, resulting in decreased topological tension, enhancing initial fork progression after re-start [6]. The unique DDN catalytic motif in the nuclease domain of Metnase is required for replication fork restart [7]. The SET histone methyltransferase domain of Metnase is also essential for fork repair and restart, but its specific role has not been defined [6].

The repair of replication forks after arrest upon DNA damage is regulated by a cascade of phosphorylation events mediated by Chk1. We discovered that Metnase is phosphorylated at S495 in response to DNA damage by Chk1 [8]. Phosphorylation of Metnase increased its DNA DSB repair capacity, but decreased its replication fork restart activity. Thus, Metnase is a downstream phosphorylation target of Chk1 that mediates the two major activities of Chk1, enhancing DNA repair and arresting replication forks [1,6,8].

In the concise study described here we found that Metnase, itself a target of Chk1, increases the half-life of total and phosphorylated Chk1 protein significantly. We show that one potential mechanism for this may be that Metnase reduces DDB1 interaction with Chk1, and decreases subsequent Chk1 ubiquitination. This represents a novel pathway of Chk1 auto-regulation, whereby one of its own targets amplifies its signal.

Human 293T cells were engineered to express wild type and S495A mutant Metnase, which lacks the Chk1 phosphorylation site, and therefore cannot be phosphorylated, as we previously described [6,8]. Metnase is ubiquitously expressed, except in cells transformed with T antigen, which is synthetically lethal with Metnase. Thus, 293T cells do not express Metnase, making them a useful model cell system to study the effects of Metnase [6-8]. V5-tagged wild type (wt) and mutant Metnase expression was confirmed by Western blot in the engineered cells. The following antibodies were used in the western and co-immunoprecipitation experiments here: FLAG, actin (Sigma, St. Louis, MO); Chk1, p317 Chk1, DDB1, ubiquitin (Cell Signaling, Danvers, MA); and V5 (Invitrogen, Carlsbad, CA).

**Figure 1:**
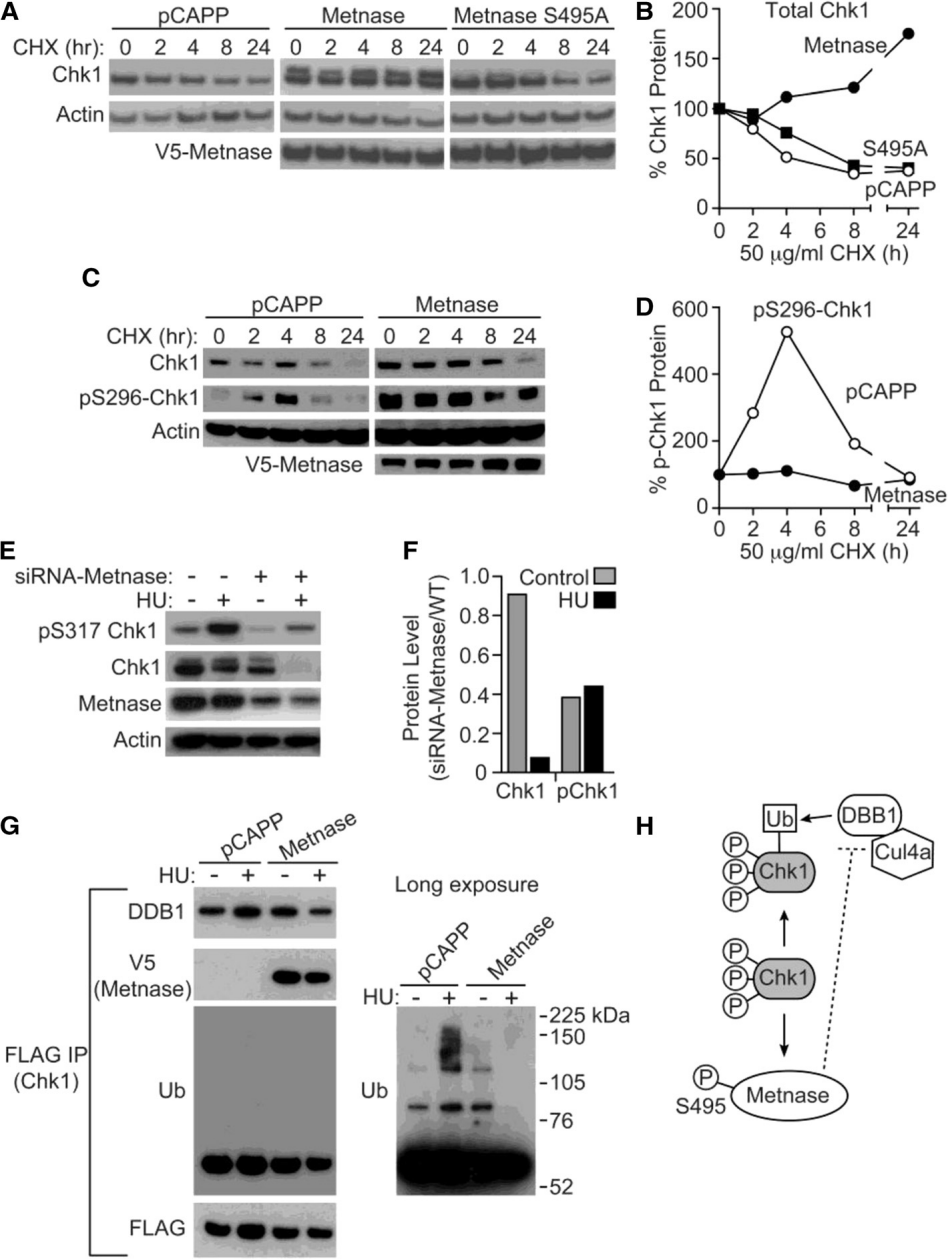
**Metnase, itself a target of Chk1 phosphorylation, stabilizes Chk1 half-life. A**, **B**- Western analysis showing Metnase stabilizes Chk1 after cycloheximide, and this is largely dependent on Chk1 phosphorylation of S495, as the S495A Metnase species, which cannot be phosphorylated, does not extend Chk1 protein half-life nearly as much. Total Chk1 levels were normalized to Chk1 present at time 0, before cycloheximide exposure. **C**,**D**- Western analysis of p296 Chk1 half-life in cells with or without Metnase (pCAPP). The presence of wt Metnase enhances the stability of phosphorylated Chk1. Phosphorylated Chk1 levels were normalized to time 0, before cycloheximide. **E**,**F**- Metnase promotes Chk1, and ATR-mediated p317 Chk1 induction after exposure to the replication stressor hydroxyurea (HU). The quantitation in panel **F** is the ratio of Chk1 or pChk1 in siRNA-Metnase cells divided by WT Metnase-expressing cells. This implies that the increase in phosphorylated Chk1 is mainly due to the increase in total Chk1 protein stability, consistent with **D**. **G**- Metnase reduces Chk1 interaction with DDB1, and reduces Chk1 ubiquitination. 293 T-pCAPP (which do not express Metnase due to T antigen) and 293 T-MET were transiently transfected with FLAG-Chk1, and treated with HU, and then FLAG was immunoprecipitated. This immunoprecipitate was probed for DDB1 and ubiquitin. Metnase over-expression reduces association of Chk1 with DDB1. HU induces Chk1 ubiquitination, but this is largely blocked by wt Metnase over-expression. A longer exposure of the ubiquitin autoradiogram is also presented. **H**- ATR phosphorylates Chk1 S317 upon replication stress. Phosphorylated Chk1 phosphorylates S495 of Metnase. Phosphorylated Metnase decreases the interaction of DDB1 with Chk1, reducing Chk1 targeting to the Cul4a E3 ubiquitin ligase, increasing Chk1 stability.

In our previous studies of the Chk1 phosphorylation of Metnase on S495 [8], we noted that cells expressing wt Metnase routinely appeared to have higher levels of Chk1. WT Metnase represents a mixture of phosphorylated and unphosphorylated Metnase species, while S495A Metnase would represent only the unphosphorylated species. The engineered 293T cell lines, expressing either wt or S495A Metnase, were exposed to cycloheximide (50 μg/mL) to arrest protein synthesis, and total Chk1 and p296 Chk1 protein levels were assessed using western analysis for protein stability, as described [1,6,8]. WT Metnase markedly increased the stability of total Chk1 protein in unstressed cells (Figure 1A,B). However, S495A Metnase, which cannot be phosphorylated by Chk1, did not appreciably alter the half-life of Chk1 protein (Figure 1A,B). In this blot, which was run longer to separate bands more efficiently, there was also an increase in dual bands of the total Chk1 protein, which likely represented total and phosphorylated Chk1 (Figure 1A). Therefore, whether wt Metnase increased the stability of phosphorylated Chk1 was examined using western analysis of cycloheximide treated cells as above. We found that wt Metnase increases the initial steady state levels of phosphorylated Chk1 (p296) at time 0, but does not alter the half-life of p296 Chk1 after cycloheximide exposure (Figure 1C,D). this gel was run for a shorter time period., thus has a single Chk1 band. The auto-phosphorylation of Chk1 at 296 is a final step in the activation of Chk1 kinase activity [8,9].

We next assessed whether Metnase repression from siRNA in 293 cells, which do express Metnase, because they are not transformed with T antigen, would also decrease Chk1 protein levels (Figure 1E,F) after replication stress. WT Metnase repression using siRNA resulted in a decrease in the steady state levels of total and p317 Chk1, and a decrease in total Chk1 after replication stress with hydroxyurea (HU, 5 mM for 4 h). ATR phosphorylates Chk1 317 after HU, which is important for initiating its auto-kinase activity, and ultimately downstream of replication fork arrest [8,9]. Thus, the presence of wt Metnase, which itself is phosphorylated by Chk1, is important for the stability of total Chk1 protein, both with and without replication stress, although the affect is greater with replication stress.

Chk1 protein stability is regulated by DDB1 targeting Chk1 to the Cul4a E3 ligase for ubquitination and destruction, and this ubiquitination is increased with replication stress [9]. In the experiments described above, wt Metnase, which is phosphorylated by Chk1, was found to increase the half-life of Chk1 protein. Therefore, we examined the interaction between DDB1 and Chk1 in the presence or absence of wt Metnase, with or without exposure to HU (10 mM for 18 h). In Figure 1E, we found that when wt Metnase was present there was less interaction of Chk1 with DDB1, especially in the presence of HU. Significantly, when wt Metnase was present, there was also a marked decrease in the ubquitination of Chk1, especially during replication stress (Figure 1G). This which would result in enhanced stability of Chk1 protein, and an increased substrate for activation by ATR [9]. These data imply that wt Metnase regulates Chk1 protein levels by decreasing its targeting by DDB1 to Cul4a for ubiquitination and proteasome destruction.

In summary, we found that Metnase stabilized Chk1 protein half-life, with or without replication stress. Upon replication stress Chk1 is phosphorylated by ATR on S317, which allows Chk1 to auto-phosphorylate, and initiate its downstream kinase cascade in the replication stress response that results in replication arrest. DDB1 down-regulates Chk1 protein levels by targeting it to the E3 ligase Cul4a for ubiquitination and destruction, even during replication stress [9]. However, the mechanism by which this destruction is regulated has not been clear. What prevents DDB1 targeting of Chk1 for destruction before the collapsed forks are repaired? This report addresses a significant unanswered question in Chk1 regulation: How are Chk1 levels maintained during replication stress? Here we demonstrate one possible answer to this question: Metnase, itself a Chk1 phosphorylation target, decreases Chk1 interaction with DDB1, and decreases subsequent Chk1 ubiquitination during replication stress (Figure 1H). Metnase serves as a switch for DDB1/Chk1 interaction, to enhance Chk1 protein stability during replication stress (Figure 1H).

We previously found that the unphosphorylated Metnase species is far better at restarting replication forks than the phosphorylated species, implying that Chk1 activity inhibited Metnase’s fork restart capability [8]. These data here, together with that previous report, suggest a layer of complexity in Chk1 signaling not currently appreciated. Chk1 phosphorylates Metnase, which decreases Metnase’s fork restart capability. Phosphorylated Metnase feeds back to stabilize Chk1 protein half-life, and therefore increases the steady state levels of phosphorylated Chk1. Phosphorylated Metnase is inefficient in the restart of collapsed replication forks, but it is recruited to DNA DSBs at higher rates, and enhances DSB repair. With Chk1-mediated Metnase phosphorylation there is further prolongation of the Chk-1 half-life, resulting in additional Metnase phosphorylation, and further reduction in replication fork restart, but further enhancement of DNA DSB repair.

Chk1 mediates cell cycle arrest after replication stress, such as occurs during chemotherapy or low oxygen conditions. This allows these cells to repair replication fork damage before they enter mitosis, which would be catastrophic if unrepaired forks persisted. Thus, Chk1 has become an important target for anti-neoplastic drug development [10]. There are several Chk1 inhibitors in clinical trials to treat cancer, and their common mechanism of action appears to be prevention of proper cell cycle arrest to allow repair of stalled or collapsed forks during replication stress, resulting in catastrophic mitosis [10]. Triple negative breast cancer is an especially appealing malignancy for treatment with Chk1 inhibitors, since these cancers have faulty replication fork repair, either from inherited or acquired BRCA1/2 deficiencies. It is possible that high Metnase levels could promote resistance to these Chk1 inhibitors by enhancing Chk1 signaling. Thus, an inhibitor of Metnase, such as one of the quinolone structures we described previously [11], could be synthetically lethal with a Chk1 inhibitor. Or, since Metnase mediates clonogenic survival to HU [6], targeting Metnase could directly cancer cell resistance to replication stressor drugs, such as camptothecin or fluorouracil. Thus, this study provides a novel target for synthetic lethality in cancers susceptible to Chk1 inhibition or replication stress.

## Competing interests

No competing interests to disclose for any author.

## Authors’ contributions

EAW generated reagents and performed experiments and analyzed data; YW performed experiments; SS performed experiments; MB analyzed data and wrote the manuscript; JW analyzed data and wrote the manuscript; S-HL analyzed data and wrote the manuscript; JAN. analyzed data and wrote the manuscript; RH conceived of the project, analyzed data, and wrote the manuscript. All authors read and approved the final manuscript.
